# How team debriefings can transform anaesthesia culture

**DOI:** 10.1016/j.bjae.2026.02.005

**Published:** 2026-04-10

**Authors:** B. Grande, M. Kolbe

**Affiliations:** 1 University Hospital Zurich, Zurich, Switzerland; 2 Simulation Centre, University Hospital Zurich, Zurich, Switzerland; 3 ETH Zurich, Zurich, Switzerland

**Keywords:** anaesthesia, debriefing, patient safety, psychological safety, team culture**s**

**Learning objectives**

After reading this article, the reader should be able to:

• Explain the purpose and structure of team debriefings in anaesthesia practice.
• Distinguish between different debriefing contexts (simulation-based, post-event and routine).
• Describe how structured debriefings can strengthen psychological safety and team learning.
• Identify common pitfalls in debriefings and strategies to avoid them.
• Outline key organisational measures to institutionalise debriefing within clinical routines.

**Key points**

• Structured team debriefings provide a framework for shared reflection and learning from both successes and failures.
• Integrating debriefings into daily anaesthesia routines promotes psychological safety, openness, collaboration, continuous improvement and transparency.
• Effective facilitation requires preparation, structure and explicit attention to hierarchy and inclusion.
• Common pitfalls include blame, dominance, lack of structure and missing closure (the ‘cliffhanger’).
• Institutional support, facilitator training and regular evaluation are essential for a sustainable culture of debriefing.

Debriefing is a structured, shared reflection practice originally developed in military and aviation and increasingly adopted within healthcare, notably in anaesthesia.^1^^,^^2^ Unlike psychological crisis interventions that focus primarily on emotional stabilization after traumatic events, structured team debriefings aim explicitly to facilitate team-based learning from recent experiences, particularly critical incidents.^3^ At the same time, routine structured debriefings can still provide valuable opportunities for peer support, helping to identify colleagues in need of additional follow-up or psychological assistance. These reflective conversations provide a protected and non-punitive environment for team members to openly explore and analyse clinical actions, decision-making processes, communication effectiveness and overall team dynamics.^4^^,^^5^ Team debriefings with the intention to learn and improve rely on evidence from team science demonstrating that structured, reflective practice among team members significantly improves team performance.^6^, ^7^, ^8^

In anaesthesia, structured debriefings contribute significantly to patients’ safety and quality improvement initiatives.^2^^,^^9^ They encourage practitioners to reflect systematically on clinical encounters, including both high-risk scenarios and routine operations. Historically, anaesthesia and broader healthcare practices have predominantly relied upon ‘Safety-I’ paradigms, focusing on identifying, examining and rectifying failures, errors or near misses to prevent future adverse events.^10^ This retrospective analytical approach, although beneficial, often overlooks valuable insights available from daily successful operations.^11^

In contrast, the Safety-II paradigm expands the scope of debriefing by emphasising that regular successful outcomes are equally valuable opportunities for learning.^11^ By examining routine procedures and standard practices, teams can better understand the factors contributing to consistent, safe care delivery.^12^ This proactive approach highlights teamwork, resilience and adaptability, which are essential components of effective anaesthesia practice. Integrating Safety-II principles enriches the learning landscape by capturing insights from everyday successes, thereby reinforcing positive clinical behaviours and decision-making patterns. Table 1 summarises the core elements of the Safety-I and Safety-II paradigms and highlights the differences between the two (Fig.1).

**Table 1:** **Comparison of Safety-I and Safety-II paradigms in anaesthesia debriefings**.

Aspect	Safety-I	Safety-II
**Primary aim**	Preventing errors and adverse events	Understanding why things go right and sustain success
**Focus**	Failures, incidents, near misses	Everyday clinical performance and adaptations
**View of people**	Seen as sources of error, variability to be controlled	Seen as resources for resilience, variability as a strength
**Change mechanism**	Corrective measures to reduce risk	Learning from positive practices to enhance resilience
**Debriefing emphasis**	Retrospective analysis of what went wrong	Reflection on what worked well and why

**Fig 1:**
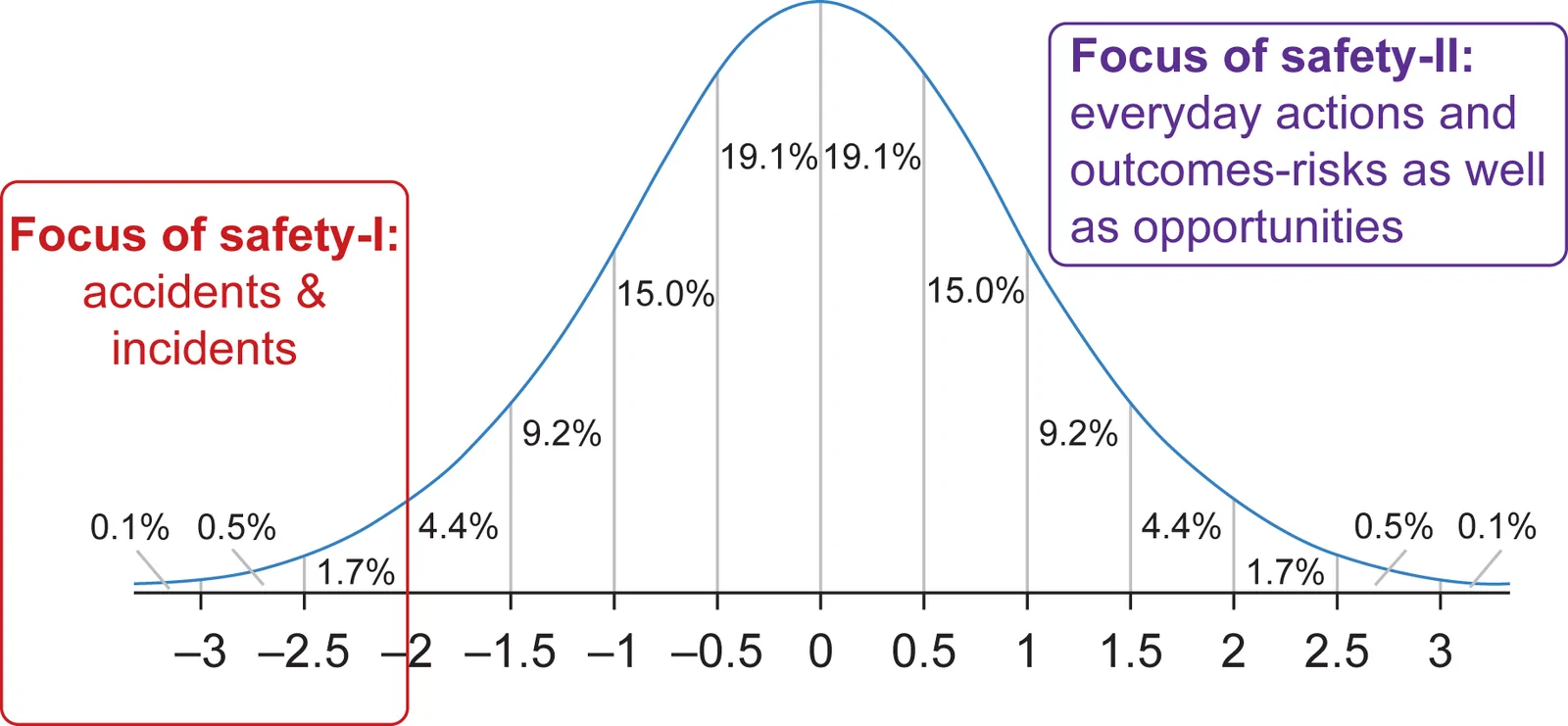
**Safety I and Safety II**.

In anaesthesia practice, debriefings take place in several contexts that differ in purpose and focus. Simulation-based debriefings are primarily educational, allowing teams to practise and reflect in a safe learning environment. Often across professions, they typically include aspects of Crew Resource Management, such as team time-outs, closed-loop communication and should rely on teamwork models.^13^, ^14^, ^15^ Real-life debriefings can occur immediately after an event (‘hot’) or after some delay (‘cold’), with hot debriefings focusing on immediate impressions and factual recall, and cold debriefings allowing for more structured reflection and system-level analysis.^16^^,^^17^ Clarifying these contexts is essential for understanding their respective contributions to team learning and cultural change in anaesthesia.

## Importance of team debriefing in anaesthesia

Anaesthesia teams routinely encounter dynamic, high-pressure situations demanding team situational awareness, precise coordination and rapid decision-making, often with limited time for reflection or discussion. Excellent collaboration profoundly influences outcomes and safety, highlighting the importance of continuous learning from each other. Team debriefings offer a structured opportunity for team members to respectfully examine how their actions, communication strategies and decision-making worked for each other (Table 2). Extensive research has demonstrated that structured debriefings are associated with notable improvements in safety outcomes, increased team morale and enhanced resilience among team members.^17^, ^18^, ^19^, ^20^, ^21^, ^22^ Debriefings also encourage interprofessional collaboration, transparency and mutual trust, ultimately fostering a proactive safety culture and allowing for discussions without fear of blame or judgment within healthcare institutions.^13^ In practice, interprofessional participation can be supported by conducting brief mini-debriefings at the end of individual cases, even if team members such as surgeons and anaesthetists do not finish their work at the same time. Moreover, operating theatre nurses can facilitate such discussions by following simple structured scripts, thereby enabling interprofessional reflection without the need for extensive formal training. By regularly integrating structured debriefing into routine clinical practice, anaesthesia teams can systematically identify areas for improvement, reinforce effective behaviours and cultivate a shared commitment toward ongoing excellence and patient-centred care.^23^ This practice not only supports individual and team growth but also contributes to broader organisational learning and systemic enhancements in the quality of care.

**Table 2:** **Elements of a structured debriefing**.

Element	Description
Set the scene	Clarify the purpose of the debriefing, establish ground rules, and build rapport.
	
Reactions phase	Allow participants to express initial emotional responses and impressions.
	
Description phase	Establish a shared understanding of what happened during the scenario.
	
Analysis phase	Explore performance, decisions, and team interactions using reflective dialogue.
	
Summary phase	Highlight key learning points and connect them to future clinical practice.
	
Application to practice	Discuss how insights can be transferred to real-world settings.

## Structuring effective team debriefings

Debriefings differ from informal team conversations in that they follow a structure.^24^ This structure is needed to ensure that the debriefing is effective. It helps team members guide the conversation and navigate common pitfalls (e.g. complaining circles, only a few team members contribute, strong hierarchy effects, avoidance of uncomfortable yet important topics).^8^ It starts before the debriefing with an appropriate preparation and setting the scene:

### Preparation and setting

Careful planning and preparation can help maximise debriefing impact and educational value. An optimal environment is characterised by a private, quiet and comfortable space that minimises interruptions, distractions and external noise, allowing team members to fully focus on reflective dialogue and learning. Selecting a conducive location not only enhances participants’ concentration but also signals respect and value for the debriefing process itself.^25^ Timing is strategically crucial; immediate debriefings capitalise on fresh recall of events and actions, enabling team members to vividly discuss and reflect upon their experiences. However, the timing must also balance immediacy with sensitivity, allowing participants sufficient emotional decompression following intense or potentially stressful clinical situations. Choosing the timing well and distinguishing routine (e.g. *postoperative*, *end-of-shift*) from prompted debriefings (e.g. *immediate*, *delayed*) is an important step; it determines what to expect, who should participate and what objectives may (not) be achieved.^17^ In addition, clearly delineated roles - including a skilled facilitator who guides the conversation, as well as participants who are willing and supported to actively engage in reflection—are used to ensure clarity, efficiency and structure throughout the session. The facilitator’s role includes creating a psychologically safe atmosphere, asking reflective questions and steering the discussion towards constructive analysis, while participants contribute openly, sharing observations, thoughts and suggestions.^26^ An effective definition of roles promotes accountability, enhances communication quality and ensures that each team member feels heard, respected and involved in the continuous improvement process. Debriefing tools help navigate the debriefing conversation.^16^^,^^17^^,^^27^, ^28^, ^29^, ^30^

#### Conducting the debriefing

Effective debriefing sessions typically follow a structured approach of multiple steps to maximise learning outcomes and enhance team learning^2^^,^^15^^,^^28^^,^^29^^,^^31^

##### Introduction

Begin by clearly articulating the objectives and scope of the debriefing, setting expectations and contributing to a psychologically safe environment that encourages open and respectful communication among all team members. This is important because team members may have very different assumptions of what a debriefing is (a way of learning) and is not (a way of punishment).^16^^,^^17^^,^^20^^,^^21^^,^^27^^,^^32^ During this initial phase, ground rules, confidentiality agreements and mutual respect should be explicitly addressed. Creating such an atmosphere allows participants to feel comfortable discussing sensitive issues openly and constructively, enhancing the quality of subsequent reflection.^33^, ^34^, ^35^

##### Reactions

Next, participants are encouraged to express their immediate responses to the recent clinical event or scenario. Allowing team members to voice initial feelings, such as frustration, relief, uncertainty, or concern, helps to alleviate emotional tension and allows potentially important debriefing topics to be revealed. In routine scheduled debriefings, this phase can often be abbreviated or adapted, as emotional reactions may play a less central role compared with post-event debriefings following adverse clinical situations. Facilitators should listen empathetically, ensuring all contributions are acknowledged and respected, thereby reinforcing trust and openness within the team.^28^

##### Description

Sometimes called ‘facts’, this step does not involve a complete, factual reconstruction of the clinical scenario. Instead, the facilitator invites the team members to briefly recount the events from their perspectives to establish a shared understanding among them of what had happened. Clarifying details at this stage is essential to resolve misunderstandings and ensure that all team members will be discussing ‘the same thing’.^28^

##### Analysis

During the analysis, the team members explore their decision-making processes, communication effectiveness and overall dynamics during the case. Facilitators guide the discussion by sharing observations and their point of view, and helping team members explore the assumptions that drove their actions by asking reflective questions.^3^^,^^31^ This may involve both strengths and areas requiring improvement.

##### Conclusions and implementation

In this step, the team summarises the key learning points and insights that emerged from the analytical discussion. Conclusions should be concise, practical and directly related to improving future practice. Recording these learning points clearly, either through written summaries or digital tools, reinforces the commitment to continuous learning and ensures lessons are not lost.^36^ Team members may also collaboratively develop specific strategies and concrete action plans to integrate the identified learning points into their future clinical practice. These plans may involve modifications to existing protocols, additional training initiatives, or improved communication strategies. Assigning clear responsibilities and timelines for implementing these strategies enhances accountability and increases the likelihood of sustained improvements in clinical practice.

## Common pitfalls and how to avoid them

Effective debriefing is crucial for learning and improving team performance, yet several common pitfalls can hinder this reflective process.^8^

### The ‘blame game’

Blame-oriented dialogue, where team members may inadvertently focus on assigning fault rather than exploring underlying causes constructively, occurs frequently.^37^ While normal and human, such an approach can trigger defensive responses, reduce openness and damage team cohesion.^25^ Facilitators may model respectful yet honest conversations, help team members explore shared contributions and focus on all team members’ psychological safety to allow for mutual learning rather than unilateral blaming.^31^

### The seniority frame

Another common pitfall is the inadequate management of hierarchical tensions within teams. Hierarchy can inhibit open communication, particularly if junior members feel intimidated or senior team members dominate discussions.^38^ Facilitators should actively flatten these hierarchies by emphasising equal participation, using debriefing tools, and encouraging contributions from all team members, regardless of seniority.^25^^,^^38^

### The truth assumption

Listening is hard, and poor listening and failure to validate team members’ experiences can disrupt effective debriefing.^39^ If participants feel unheard or undervalued, they may disengage from future discussions.^26^ Facilitators are well advised to reflect on their own listening approach as well as to model active listening, validate contributions meaningfully and ensure all perspectives are acknowledged to maintain engagement.^26^^,^^31^

### The ‘let’s just talk’ approach

While following a debriefing structure may seem awkward at first, it is essential for debriefing effectiveness. A lack of structure can lead to unfocused discussions, complaining circles, and even manifest unhelpful team dynamics. A clearly defined and commonly shared structure with explicit steps help all team members to contribute and learn from each other.^40^^,^^41^

### The ‘cliffhanger’

Another common pitfall is the so-called ‘cliffhanger,’ where a debriefing ends without exploring take-away messages, summarising key insights, or clarifying the next steps. This lack of closure can leave team members uncertain about what has been learned, what to conclude and how improvements will be implemented. Facilitators should therefore always ensure that debriefings conclude with a clear summary of take-home messages and concrete action points.^3^

By anticipating and addressing these common pitfalls, facilitators can significantly enhance the quality and outcomes of debriefings.

## Psychological safety in debriefings

Central to the success of any effective debriefing session is the establishment of psychological safety as the shared belief among team members that the team is safe for interpersonal risk-taking: everybody can speak their mind.^35^ Importantly, psychological safety is not about being agreeable, nice, or polite; rather, it represents a critical climate in which team members feel confident to speak openly, voice concerns, acknowledge uncertainties and admit mistakes without fear of embarrassment, punishment or rejection.^35^

Within healthcare settings, especially during debriefings after high-stakes clinical scenarios, psychological safety significantly shapes the depth, honesty and effectiveness of reflective discussions.^1^^,^^16^^,^^17^^,^^23^^,^^34^^,^^42^^,^^43^ Debriefings conducted in psychologically safe environments allow participants to openly explore challenging situations, facilitating collective learning, problem-solving and ultimately enhancing patients' safety.^33^ Conversely, an absence of psychological safety discourages team members from discussing errors or potential improvements, thereby limiting learning and perpetuating suboptimal practice.^8^

Facilitators play an instrumental role in cultivating psychological safety. Edmondson’s research indicates that leaders who demonstrate genuine curiosity, humility and openness substantially enhance psychological safety within teams.^35^ Effective debriefing facilitators actively promote balanced participation, ensuring that all team members—regardless of their role or seniority—feel equally valued and respected. Facilitators must proactively counteract hierarchical barriers by explicitly inviting input from less experienced or less vocal participants, validating their contributions and preventing dominant personalities from overshadowing others.^34^^,^^42^ Skilled facilitators adeptly manage conflict and sensitive emotional reactions.^44^ They model empathy, active listening and impartiality, creating an atmosphere where emotional vulnerability is supported rather than suppressed. By recognising and responding sensitively to non-verbal cues—such as body language, facial expressions or hesitations—facilitators ensure that emotional discomfort or distress is addressed constructively.^44^ Additionally, structured communication strategies, such as combining advocacy with inquiry, appreciative inquiry and open-ended questioning, further strengthen psychological safety.^31^^,^^42^ Appreciative inquiry involves framing discussions positively, emphasising strengths and potential solutions rather than dwelling solely on deficiencies or failures.^26^ Open-ended questions encourage deeper reflection and genuine dialogue, inviting team members to articulate their experiences and reasoning processes without judgment.

Facilitators should be trained in these communication and observation techniques, reinforcing their capacity to uphold psychological safety consistently. Structured facilitator training programmes that emphasise Edmondson’s principles (openness, curiosity, humility and mutual respect) enhance facilitators’ skills, improving the overall quality of debriefings.^26^^,^^35^

## Practical aspects

Ensuring routine and effective debriefings requires deliberately embedding these practices within an institution’s broader organisational culture, structures and clinical workflows.^1^ This integration goes beyond merely scheduling periodic discussions; it involves creating an environment where reflective practice is viewed as a normative aspect of clinical work and continuous improvement. Organisational leadership plays a crucial role by advocating for the consistent use of debriefing practices and visibly supporting their value. When leaders emphasise the importance of reflective learning and openly participate in these sessions, it signals a robust institutional commitment, thereby fostering broader acceptance among staff members.^2^^,^^8^^,^^45^ Several practical recommendations can facilitate successful institutional integration. We acknowledge that facilitator training may be resource-intensive; therefore, cost-effective formats such as peer mentoring, short workshops or blended online modules may be valuable alternatives to ensure sustainability.

### Developing a shared understanding

Firstly, clearly articulated institutional protocols and guidelines should be developed and communicated across all levels. These protocols help standardise debriefing practices, ensuring consistency, clarity and transparency in objectives, processes and expected outcomes. They also delineate roles, responsibilities and accountability measures, thereby reducing ambiguity and enhancing participation. Furthermore, such guidelines serve as tangible references, empowering staff to confidently conduct or engage in debriefings.

### Building capacity

Secondly, institutions should implement dedicated facilitator training programmes aimed at developing advanced facilitation skills. Such training programmes should be evidence-based and comprehensive, covering key skills including active listening, psychological safety management, conflict resolution and effective questioning techniques.^3^^,^^17^^,^^20^ Well-trained facilitators, who are able to help decide when and how debriefings are useful, can significantly enhance the effectiveness of debriefings by ensuring discussions remain productive, respectful and aligned with institutional goals. Regular refresher courses and ongoing mentorship opportunities further sustain facilitator expertise and motivation.

### Feedback

Thirdly, creating structured feedback mechanisms is essential for maintaining a continuous cycle of learning and improvement. Institutions should systematically collect data and feedback on the effectiveness of debriefings, facilitator performance and participant satisfaction. Regular evaluation helps identify strengths and areas for improvement, promoting a culture of accountability and continuous development. Transparent feedback systems reinforce the institution’s commitment to quality improvement and empower staff to actively engage in refining the debriefing process.

### Embedding debriefings into daily clinical routines

Finally, embedding regular debriefings into daily clinical routines reinforces their importance, normalises reflective practices and supports sustained professional growth. Making debriefings habitual rather than occasional ensures their lasting presence within the institutional culture, enhancing team resilience, collaboration and overall safety for patients. Allocating protected time, resources and physical space for routine debriefings demonstrates organisational investment and ensures practical barriers to participation are minimised.

## Conclusions

Structured debriefing has emerged as a cornerstone of learning and quality improvement within anaesthesia and broader healthcare practice. Initially developed in high-reliability sectors such as aviation and the military, debriefing has since been effectively adapted to the clinical context, where dynamic, high-pressure environments necessitate agile team coordination, transparent communication and rapid decision-making. As the evidence demonstrates, when debriefings are consistently structured, facilitated by trained individuals and embedded within a culture of psychological safety, they offer an unparalleled opportunity for collective reflection and learning. This practice not only enables teams to analyse adverse events and near misses (as emphasised by the traditional Safety-I paradigm) but also allows them to explore successful outcomes in routine care—an essential element of the Safety-II approach. The dual focus enriches learning by encouraging deeper insight into both what goes wrong and what consistently goes right, thereby reinforcing adaptive behaviours and promoting resilience in clinical practice.

Structured debriefings serve a dual purpose: they function as both a learning mechanism and a cultural intervention. By creating a safe space for open dialogue, they nurture psychological safety, flatten hierarchical barriers and validate the contributions of all team members. To ensure sustained benefit, healthcare institutions must incorporate debriefing as a routine practice. This involves clear protocols, dedicated facilitator training, regular feedback mechanisms and integration into daily clinical workflows. With intentional design and ongoing support, debriefing can evolve from an occasional educational exercise into a transformative organisational practice that enhances team performance, nurtures professional growth and, ultimately, improves patients’ safety across the system.

Ultimately, structured team debriefings do more than improve technical performance or safety: they have the power to reshape the culture of clinical practice, and foster openness, trust and continuous collective learning as defining features of daily practice.

## MCQs

The associated MCQs (to support CME/CPD activity) are accessible at www.bjaed.org/cme/home for subscribers to BJA Education.

## Declaration of interests

The authors declare that they have no conflicts of interest.
